# Neonatal pneumococcal colonisation caused by Influenza A infection alters lung function in adult mice

**DOI:** 10.1038/srep22751

**Published:** 2016-03-04

**Authors:** Meaghan FitzPatrick, Simon G. Royce, Shenna Langenbach, Jonathan McQualter, Patrick C. Reading, Odilia Wijburg, Gary P. Anderson, Alastair Stewart, Jane Bourke, Steven Bozinovski

**Affiliations:** 1 RMIT University, School of Health Sciences and Health Innovations Research Institute, AUSTRALIA; 2 The University of Melbourne, Lung Health Research Centre, Department of Pharmacology & Therapeutics, Parkville, AUSTRALIA; 3 The WHO Collaborating Centre for Reference and Research on Influenza, Victorian Infectious Diseases Reference Laboratory, at The Peter Doherty Institute for Infection and Immunity, Melbourne, AUSTRALIA; 4 The University of Melbourne, Department of Microbiology and Immunology, at The Peter Doherty Institute for Infection and Immunity, Melbourne, AUSTRALIA; 5 Department of Pharmacology, Monash University, Clayton, Victoria, AUSTRALIA

## Abstract

There is emerging epidemiological data to suggest that upper respiratory tract bacterial colonisation in infancy may increase the risk of developing respiratory dysfunction later in life, and respiratory viruses are known to precipitate persistent colonisation. This study utilized a neonatal mouse model of *Streptococcus pneumonia* (SP) and influenza A virus (IAV) co-infection, where bronchoalveolar leukocyte infiltration had resolved by adulthood. Only co-infection resulted in persistent nasopharyngeal colonisation over 40 days and a significant increase in airway resistance in response to *in vivo* methacholine challenge. A significant increase in hysteresivity was also observed in IAV and co-infected mice, consistent with ventilatory heterogeneity and structural changes in the adult lung. Airway hyper-responsiveness was not associated with a detectable increase in goblet cell transdifferentiation, peribronchial smooth muscle bulk or collagen deposition in regions surrounding the airways. Increased reactivity was not observed in precision cut lung slices challenged with methacholine *in vitro*. Histologically, the airway epithelium appeared normal and expression of epithelial integrity markers (ZO-1, occludin-1 and E-cadherin) were not altered. In summary, neonatal co-infection led to persistent nasopharyngeal colonisation and increased airway responsiveness that was not associated with detectable smooth muscle or mucosal epithelial abnormalities, however increased hysteresivity may reflect ventilation heterogeneity.

Influenza-associated hospitalization rates in children younger than 5 years old are exceeded only by the elderly^1^. Globally, it has been estimated that around 20 million cases of influenza infections occur worldwide in children younger than 5 years, which accounts for 13% of paediatric cases of acute lower respiratory tract infections^2^. Infants aged less than one year have been reported to be even more susceptible to influenza-associated hospitalizations^3^, which is consistent with children under the age of 6 months being too young for vaccination. The clinical response to different viral strains can vary from mild disease to severe pneumonia, and immunological and inflammatory profiles have been extensively characterised, where pandemic strains are more likely to initiate an exaggerated inflammatory response that culminates in severe lung injury^4^.

Young children also represent a major reservoir for *Streptocococcus pneuemoniae* (SP) carriage^5^, which is the predominant microbe responsible for bacterial pneumonia. Although there are multiple environmental, host and microbiological factors that increase an individual’s susceptibility to pneumococcal disease, infection with IAV in particular, has been associated with an increase in pneumocococcal burden^6^. Notably, concurrent IAV infection is essential for the transmission of SP from colonised mice to their naive co-housed littermates, as transmission could be prevented by inhibiting viral replication^7^. The detection of bacteria that can colonise the airways occurs frequently during significant bronchiolitis episodes; hence the common terminology of viral wheeze may not accurately reflect the aetiological nature of many acute events in children under the age of 5 years^8^,^9^. Neonatal upper airway colonisation is also associated with increased risk of bronchiolitis in early life independent of concurrent asthma^10^. There are also emerging epidemiological data to suggest that asymptomatic nasopharyngeal bacterial colonisation is associated with persistent wheeze in children, where the prevalence of asthma at 5 years of age was significantly increased in the children colonised as neonates in the hypopharyngeal region with microbes including SP^11^.

It has also been established that influenza and other viruses can acutely alter lung function and lead to airway hyper-responsiveness (AHR) in response to increasing concentrations of constrictor agonists, such as methacholine (MCh)^12^. Using a low-frequency, forced oscillation technique, heightened responsiveness to both inhaled and intravenous MCh has been observed during the acute phase of influenza A virus (IAV) infection in mice. In this study, no intrinsic smooth muscle defect was observed, but rather an increase in permeability of the alveolar–capillary barrier was facilitating greater MCh access to airway smooth muscle^13^. Furthermore, increased AHR was shown to be transient in nature as lung function normalised with resolution of inflammation and tissue injury in adult mice^13^. In contrast to adult mice, physiological and inflammatory responses to IAV infection were assessed in infant mice, where residual AHR persisted for 21 days when inflammation and alveolar–capillary permeability had fully resolved^14^. In the current study, the effect of infant co-infection with SP and IAV on adult mouse lung function was assessed, revealing persistence of MCh-induced hyper-responsiveness that was independent of mucosal epithelial abnormalities.

## Methods

### Animals and Model of Neonatal Co-infection

Advanced pregnant C57BL/6 dams were obtained from Animal Resources Centre, Western Australia. Dams were housed separately and monitored for births, with minimal disruption. Upon birth, dams were housed with their litters in light-protected cages until weaning at 3–4 weeks of age. Mice were housed at 22 °C under normal 12 : 12 h light : dark cycle, and given free access to a normal diet and water, and monitored for signs of illness or distress. All experimental procedures performed in mice were approved by the Animal Ethics Committee of the University of Melbourne (approval #1111986 & #1413288), complied with the National Health and Medical Research Council (NHMRC) Australian Code of Practice and were carried out in accordance with the approved guidelines. Neonatal mice were infected with SP and/or IAV as previously described^7^ with the following minor modifications. At 5 days of age, C57BL/6 mice were infected intranasally without anaesthesia with vehicle (sterile PBS) or SP (serotype 19F/EF3030, 2 × 10^3^ CFU/mL) in a volume of 3 μL sterile PBS. At 12 days of age, mice were infected intranasally with vehicle (sterile PBS) or IAV (strain HK × 31, H3N2, 7 × 10^3^ PFU/mL) in a volume of 3 μL sterile PBS.

### Collection and Measurement of Specimens

Following neonatal challenge with SP and/or IAV, mice were allowed to recover and assessed starting at 6–8 weeks of age in early adulthood. At time of analysis, mice were euthanized by overdose of sodium pentobarbitone and bronchoalveolar lavage (BAL) was performed by tracheotomy and total and differential BAL cell counts determined as previously described^15^. Briefly, at time points prior to 6–8 weeks, nasopharyngeal and lung tissues were collected, homogenised and serial dilutions of tissue homogenates were cultured on horse blood agar plates to determine bacterial load as previously described^7^.

### Measurement of Airway Hyperresponsiveness *in vivo* and *in vitro*

Lung function was measured using a modification of the low-frequency forced oscillation technique and a small-animal ventilator (flexiVent; Scireq, Montreal, QC, Canada) as described^13^. Respiratory impedance (Z_rs_) was measured and partitioned into airway and parenchymal components to allow calculation of Newtonian resistance (Rn, equivalent to airway resistance (Raw) because of the high compliance of the chest wall), tissue damping (G) and elastance (H), as previously described^14^. Airway inertance (I_aw_) is negligible after correcting for the tracheal cannula and is not reported. Tissue hysteresivity (η) was calculated as the ratio G/H^16^. Baseline respiratory mechanics was recorded, followed by saline aerosol and increasing doses of MCh delivered by an ultrasonic nebuliser (MCh; 1, 3, 10, 30, 100 mg/mL). Differences in responsiveness were assessed as the maximum responses to 30 mg/mL MCh challenge. *In vitro* small airway reactivity in response to MCh was assessed via the precision cut lung slice technique as previously described^17^. Briefly, low-melting point agarose (GIBCO/Invitrogen, Australia) was used to inflate lungs, sequential slices (150 μm) using a vibratome (VT 1000 S, Leicamicrosystems) were cut and lung slices containing intrapulmonary airways ranging from 150–300 μm in diameter were incubated at 37 °C and 5% CO_2_ for 24 h prior to use in experiments.

### Histology and Immunohistochemistry

The left lobe of lung was removed *post mortem* and immediately fixed in 10% neutral-buffered formalin. Tissues were paraffin-embedded and cut at a thickness of 5 μm. Sections were stained with H&E, Masson trichrome for assessment of epithelial and sub-epithelial collagen content or with Alcian blue–periodic acid-Schiff for assessment of goblet cell transdifferentiation. Morphometric evaluation of lung tissue sections was performed as described previously^18^. A minimum of five bronchi selected according to size (150 to 350 mm luminal diameter) were analysed per mouse. To assess the presence of airway smooth muscle bulk, primary antibody to anti-α smooth muscle actin (α-SMA, Dako, Glostrup, Denmark) was used and the thickness of labelled α-SMA per length of basement membrane measured in a minimum of 5 bronchi, as previously described^19^. Images of stained slides were captured using Aperio ImageScope software.

### Quantitative Real-time PCR for gene expression analysis of lung tissue

RNA was purified from whole lung tissue using RNeasy kit as per manufacturer’s instructions and cDNA was prepared as previously described^20^. Taqman PCR primers were used to perform Q-PCR, where all threshold cycle values (Ct) were normalized to control (glyceraldehyde phosphate dehydrogenase) and the relative fold change determined by the ΔΔCt value as previously described^20^.

### Data analysis

Data and presented as the mean ± standard error of mean (SEM) for *n* mice. All data were statistically analysed using GraphPad Prism 5.0 (Graphpad, San Diego, CA). Where detailed and appropriate, one-way analyses of variance (ANOVA) with Bonferroni or Dunnett’s post-hoc tests were used. In other cases, unpaired Student’s t-tests were used to analyse data. p < 0.05 was considered to be statistically significant.

## Results

### Neonatal co-infection facilitates long term nasopharyngeal SP colonisation

**Figure 1:**
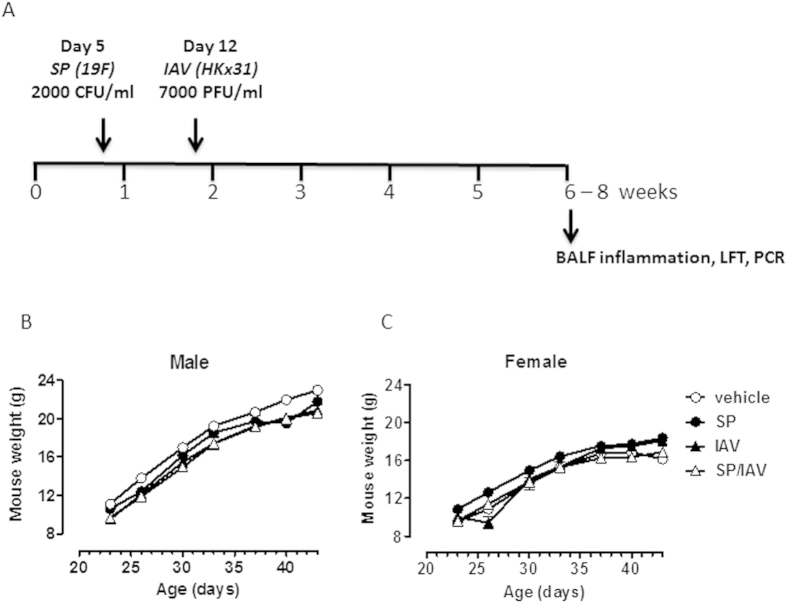
Model of neonatal respiratory co-infection. (**A**) Neonatal mice were infected with Streptococcus pneumonia (SP) at 5 days of age (or PBS vehicle) and co-infected with influenza A virus (IAV) at 12 days of age (or PBS vehicle, resulting in 4 treatment groups (vehicle, SP, IAV, SP/IAV). Exposure to infections in infancy did not alter somatic growth in male (**B**) or female (**C**) mice from age of weaning into adulthood (n = 4−9 representative of infant mice). LFT; lung function testing, BALF; bronchoalveolar lavage fluid.

**Figure 2:**
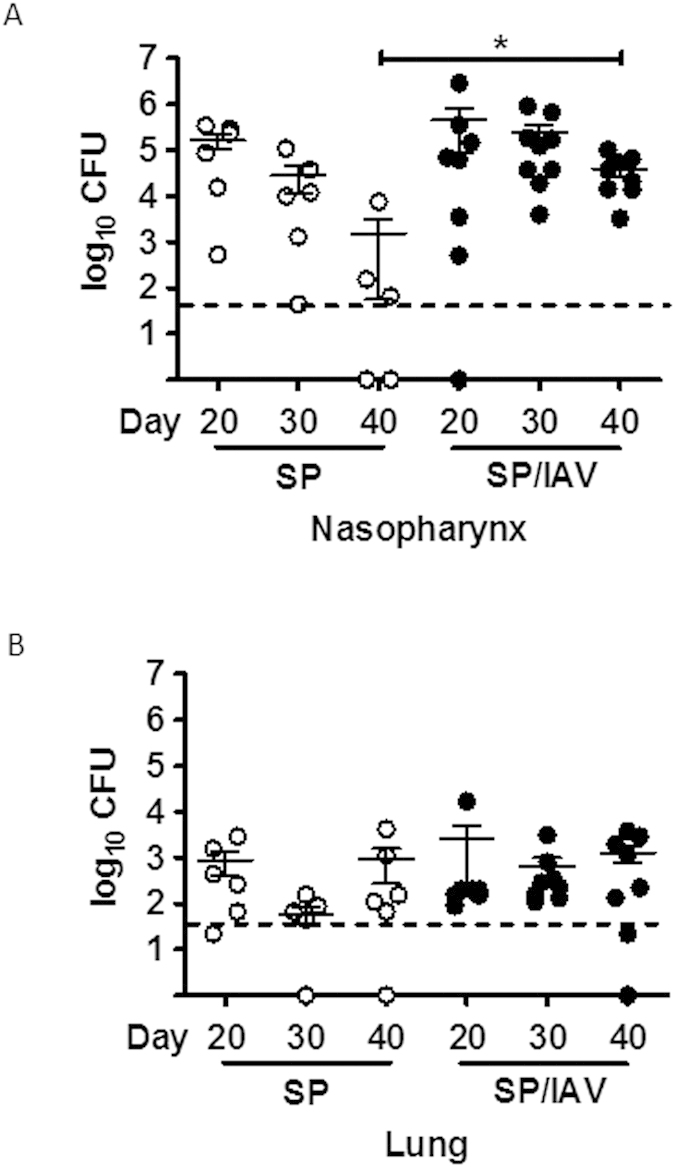
Persistent upper airway colonisation following neonatal co-infection. Bacterial burden in the upper airways (nasopharynx, (**A**)) and lower airways (whole lung, (**B**)) was assessed at 20, 30 and 40 days of age in SP and SP/IAV treated mice (n = 6−9). Data are expressed as total colony-forming units in whole lung tissues that were homogenised and grown on horse-blood agar plates. *p < 0.05 compared to day 40 SP alone.

An established co-infection model was used, where IAV is known to disrupt clearance of SP leading to a transient increase in nasopharyngeal and lower airway bacterial carriage in adult mice^7^. In our study, neonatal mice were infected and outcomes were assessed in early adulthood as described in Fig. 1A. Early life infection with SP and/or IAV did not have any long lasting effects on body weight of male and female mice across all groups into adulthood (Fig. 1B,C). To determine the chronicity of SP carriage in the upper and lower airways, a detailed kinetic study was performed. Bacterial load in nasopharyngeal (Fig. 2A) and lung (Fig. 2B) homogenates were assessed at time points of 20, 30 and 40 days of life. In neonatal mice infected with SP alone, transient nasopharyngeal carriage steadily declined to negligible levels by day 40 of life. This is in contrast to co-infected neonatal mice, where levels of SP upper airway carriage persisted over the kinetic study leading to a significant 1-log increase in load at Day 40 relative to mice infected with SP alone (Fig. 2A).

**Figure 3:**
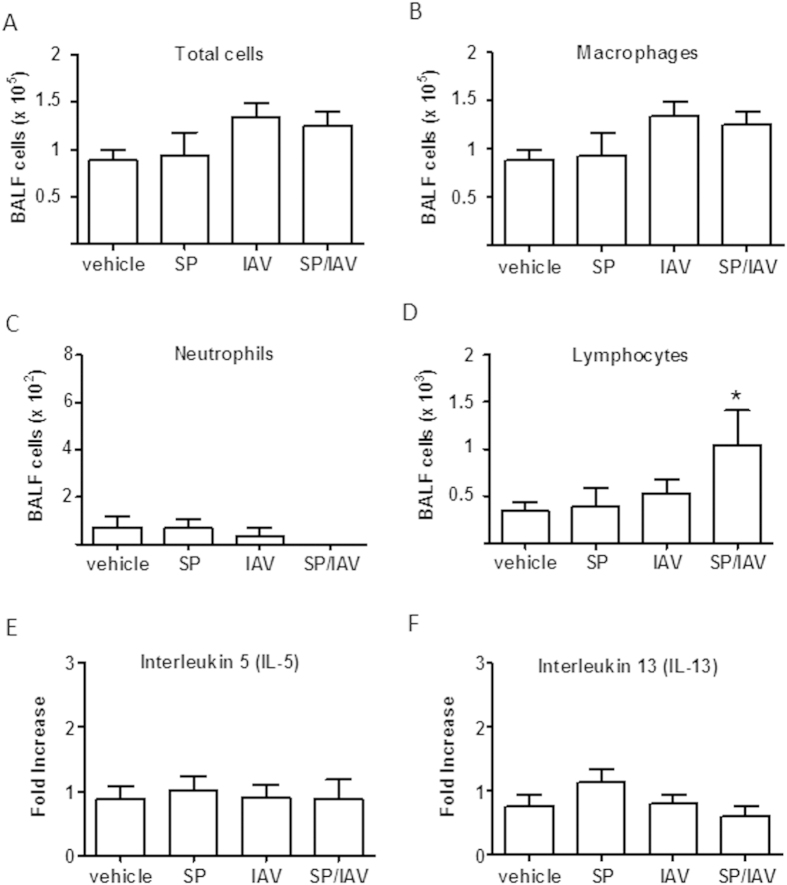
Neonatal co-infection does not elicit T_H_2 immune response in adult lung. Bronchoalveolar lavage fluid from adult mice exposed to infant co-infection was assessed for (**A**) total and differential (**B**) macrophage, (**C**) neutrophil and (**D**) lymphocyte cell counts (n = 5−11). Taqman PCR analysis for gene expression of T_H_2 cytokines (**E**) interleukin-5 and (**F**) interleukin-13 in lung tissue is expressed as fold-change compared to vehicle-treated control and normalised to GAPDH housekeeping gene (n = 6). *p < 0.05 compared to vehicle lymphocytes. BALF; bronchoalveolar lavage fluid.

In this model, there was no evidence for significant invasive dissemination of SP into the lower airway (Fig. 2B), suggesting that the bacteria localised predominately to the upper airways. In addition, the number of inflammatory leukocytes in the BAL compartment was assessed in adult mice following infection during neonatal phase of life. The data is indicative of the inflammatory process being fully resolved in response to IAV and/or SP as total, neutrophil and macrophages numbers were not altered (Fig. 3A–C). There was a small but significant increase in lymphocyte counts in co-infected mice compared to vehicle control (Fig. 3D). To determine whether the increase in BAL lymphocyte numbers were associated with an increase T_H_2 cytokines in the lung, QPCR was performed on IL-5 and IL-13, where no increase in expression was observed (Fig. 3E,F).

### Co-infection with SP and IAV increased central airways resistance and hysteresivity

**Figure 4:**
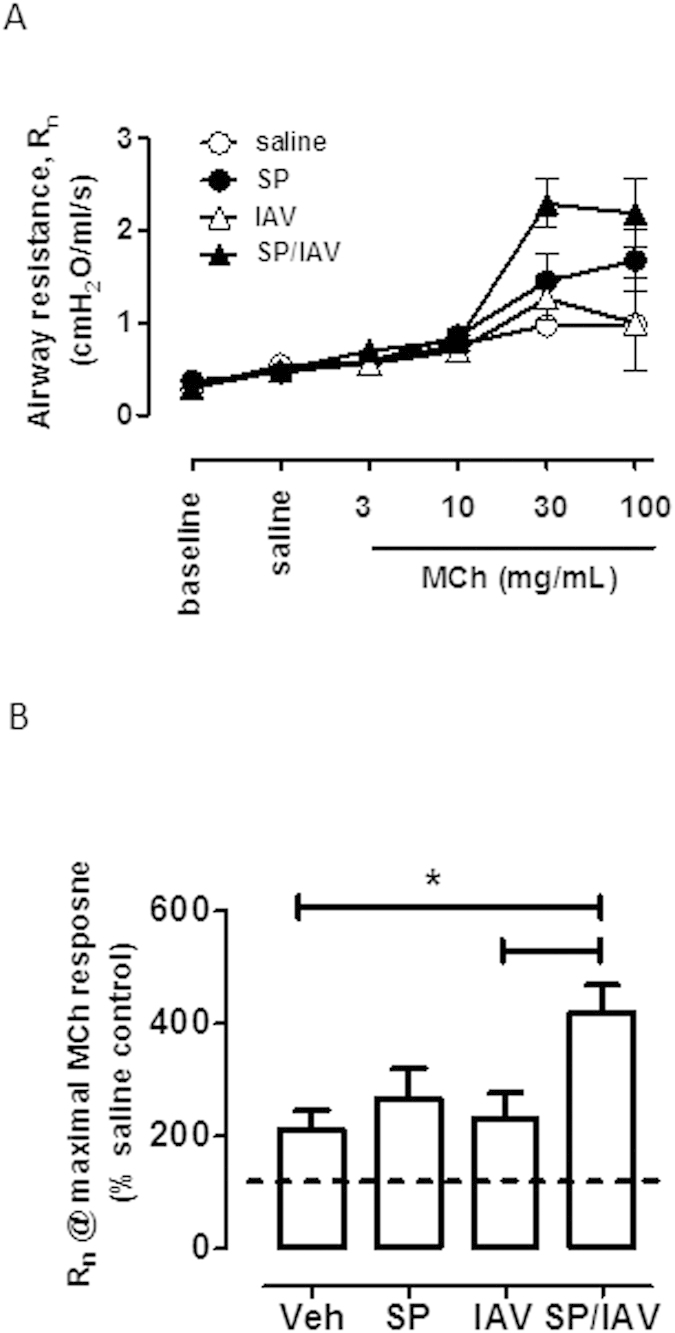
Increased airway resistance *in vivo* following neonatal co-infection. (**A**) Newtonian resistance (Rn), which is representative of central airways resistance was assessed via SCIREQ® Flexivent small-animal ventilator. Nebulised doses of methacholine (MCh) in injectable saline were delivered via tracheal cannula. (**B**) Maximal Rn response to MCh is expressed as % saline control response (n = 5−12). *p < 0.05 compared to vehicle and to IAV treated mice.

**Figure 5:**
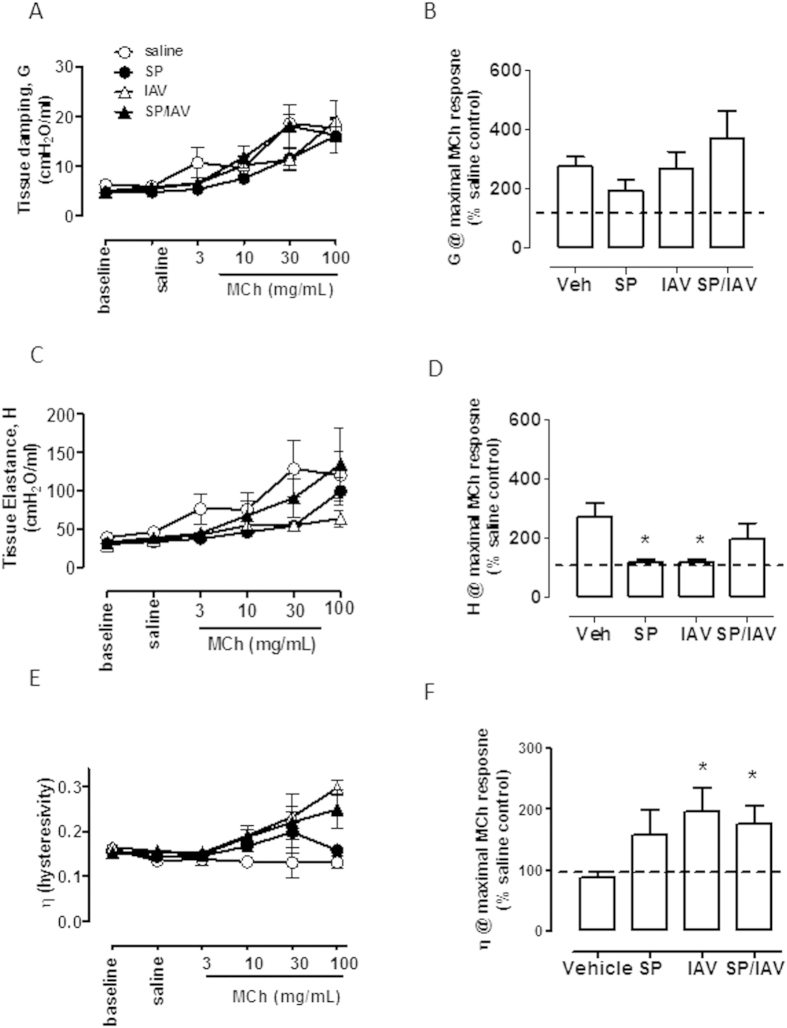
Increased airway hysterestivity following neonatal co-infection. Tissue damping (*G*; (**A**,**B**)), tissue elastance (*H*; (**C**,**D**)) and tissue hysterestivity (η; (**E**,**F**)) were also assessed with SCIREQ® Flexivent in response to nebulised doses of MCh (n = 5−12). *p < 0.05 compared to vehicle treated mice.

In mice co-infected with SP and IAV as neonates, baseline lung function responses were not different to that of vehicle treated mice. However newtonian resistance (Rn), which is equivalent to central airway resistance, increased at the higher doses of MCh, peaking at 30 mg/mL (Fig. 4A). MCh responses at 30 mg/mL were compared across all four groups and presented as a percentage change above nebulised saline response. The data demonstrates a significant increase (p < 0.05) in central airway resistance (Rn) only in co-infected mice that was 2-fold higher than mice treated with vehicle as neonates (Fig. 4B). Baseline G (tissue damping), H (tissue elastance) and hysteresivity (G/H) were also assessed in adult mice treated with SP and IAV or vehicle in infancy. These additional measures of lung function also showed no difference at baseline (Fig. 5A,C,E). Infection in infancy did not significantly affect MCh-induced increases in G (Fig. 5A,B), The significant increase in MCh-induced H detected in vehicle and co-infected mice was absent in mice treated with either SP or IAV alone (Fig. 5C,D). Hysteresivity (η = G/H), which has been used to characterise tissue mechanics was shown to increase with increasing concentration of MCh in co-infected mice above vehicle treated mice (Fig. 5E). Maximal MCh responses across all four groups revealed a significant increase in η in both IAV and SP/IAV treated mice (Fig. 5F).

### Assessment of airway remodelling in adult mice co-infected as neonates

**Figure 6:**
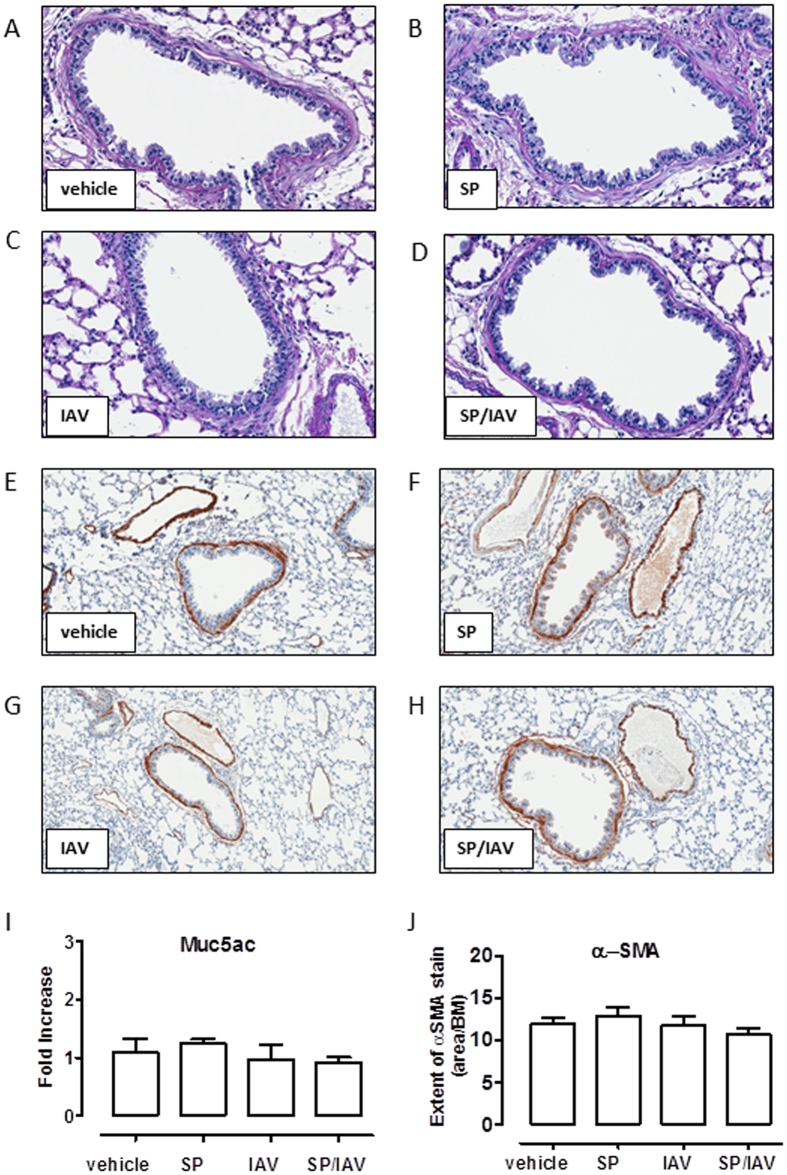
Neonatal co-infection does not induce mucus hyper-secretion or increased airway smooth muscle bulk. Representative images of immunohistochemistry shows Ab-PAS (**A**–**D**) and α-smooth muscle actin (α-SMA; (**E**–**H**) staining of lung tissue from adult mice exposed to infection in early life. (**I**) Taqman PCR analysis of mucin marker Muc5ac gene expression in lung tissue is expressed as fold-increase compared to vehicle-treated control and normalised to GAPDH housekeeping gene (n = 6). (**J**) Quantification of airway α-SMA staining (brown) is expressed as area of stain/basement membrane (BM) length (n = 5−11).

**Figure 7:**
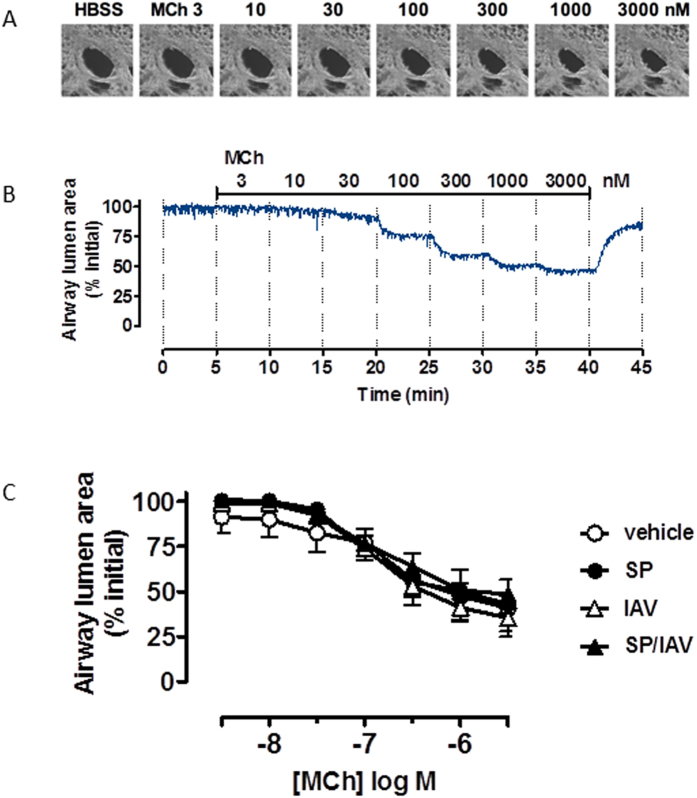
Neonatal co-infection does not elicit hyperreactivity *in vitro* in lung slices. (**A**) Representative phase-contrast images and (**B**) time-course trace of an intrapumonary airway in mouse precision cut lung slice contracting to increasing concentrations of perfused methacholine (MCh). Time-course trace and (**C**) grouped data are expressed as % inital airway lumen area as calculated following greyscale analysis of airway lumen size (n = 3−5).

**Figure 8:**
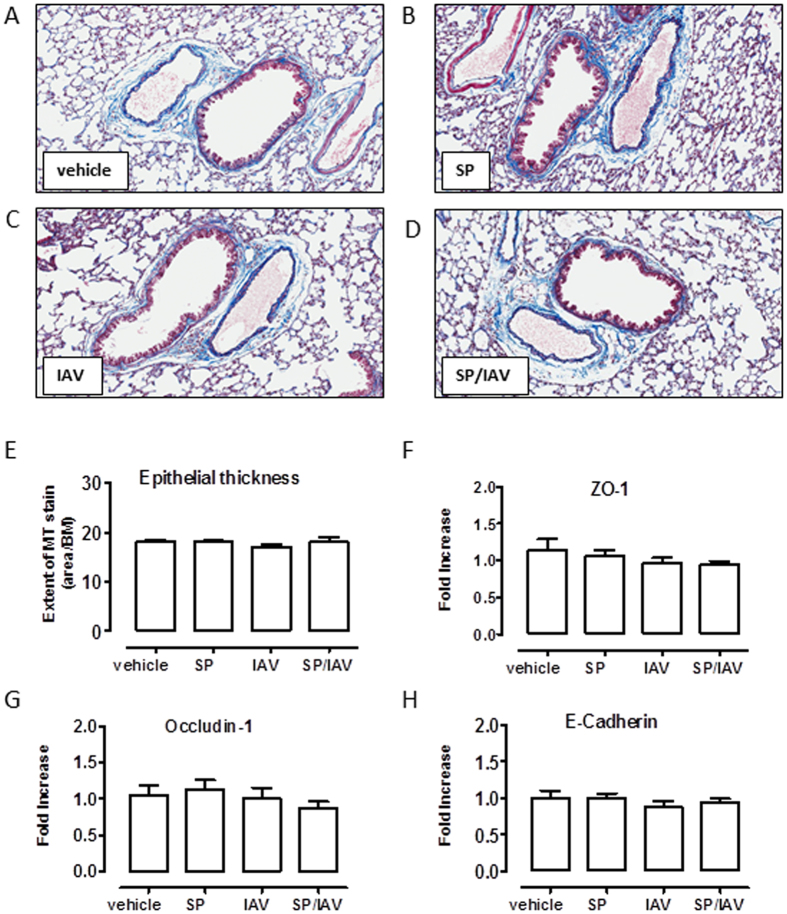
Neonatal co-infection is not associated with airway epithelial disruption. Representative histological images (**A**–**D**) of Massons Trichrome (MT) staining of lung tissue from mice exposed to infection in early life. Quantification of epithelial thickness (**E**) is expressed as area of MT stain (purple)/basement membrane length (n = 5−11). Taqman PCR analysis of epithelial integrity markers (**F**) ZO-1, (**G**) occludin-1 and (**H**) E-Cadherin are expressed as fold-increase compared to vehicle-treated control and normalised to GAPDH housekeeping gene (n = 6).

To assess whether altered airway responsiveness was due to remodelling processes occurring in the lung, mucus and smooth muscle was quantified histologically and via gene expression analysis of lung tissue. AB-PAS staining revealed no observable increase in goblet cell numbers across all groups (Fig. 6A–D), and the lack of goblet cell expansion was consistent with unchanged Muc5ac mucin gene expression (Fig. 6I). Immunohistochemical assessment of α-smooth muscle actin positive area surrounding airways expressed relative to basement membrane area revealed no significant difference across the treatment groups (Fig. 6J). In addition*, in vitro* airway reactivity was investigated in precision cut lung slices. Consistent with the absence of increased area for α-smooth muscle *in vivo*, contractile responses of size-matched intrapulmonary airways to MCh *in vitro* were unaltered between all treatment groups, where MCh caused a ~60% reduction in airway lumen area in all mice tested (Fig. 7A–C). Since mucin or smooth muscle abnormalities, were not detected, we considered the potential for epithelial disruption contributing to increased airway responsiveness *in vivo.* Histologically, the epithelium appeared normal in all treatment groups and when quantified, epithelial thickness measures were comparable (Fig. 8A–E). Gene expression analysis of epithelial integrity markers including ZO-1, Occludin-1 and E-cadherin also yielded no significant differences across the treatment groups relative to vehicle treated mice, although there was a small trend towards a reduction in ZO-1 and Occludin-1 in co-infected mice (Fig. 8F–H). In addition, total protein concentration was determined in the BALF as an alternative marker for barrier integrity. BALF protein levels in vehicle (538 ± 40), SP (565 ± 22), IAV (546 ± 15), SP + IAV (511 ± 36) μg/mL were not significantly different across the challenge groups. Additionally, FACS analysis whole lung tissue (Supplementary Figure S1) revealed no significant difference in the relative abundance of EpCAM^+^ MHCII^+^ AT2 cells in the non-hematopoietic (CD45^−^), non-endothelial (CD31^−^) cell fraction, which further demonstrates that there was no alteration in epithelial cell composition across the treatment groups. This data was consistent with no observed increase in surfactant protein C and D expression, as assessed by QPCR (supplementary figure 2).

## Discussion

In this study, a significant increase in central airways resistance in response to MCh challenge was only observed in adult mice co-infected with SP and IAV during infancy, in contrast to mice infected with a single respiratory pathogen. In a previous study, residual lung function defects in adulthood were observed in mice that were challenged with IAV in infancy^14^. The differential response to IAV alone may reflect mouse and viral strain differences, where Balb/c mice inoculated with Mem/71 were previously been used, in contrast to this study where C57BL/6 mice infected with HKx31 were assessed. Histologically, there was no evidence for remodelling of the airways, with no increase in goblet cells or Muc5ac expression or increase in smooth muscle bulk. Functionally, airway smooth muscle appears to be normal as assessment of MCh constrictor responses in isolated lung slices were not altered in co-infected mice.

Since IAV-induced disruption of the airway-epithelial barrier has previously been reported to promote AHR due to increased MCh accessibility to smooth muscle, we also assessed epithelial integrity. Co-infection with virulent strains of IAV and SP has been shown to lead to greater loss of basal epithelial cells and poor re-establishment of the normal airway epithelium during reparative processes^21^. However, histologically there was no evidence for disruption of lower airway epithelial barrier surrounding airways, and transcript expression of the epithelial integrity markers (ZO-1, occludin-1 and E-cadherin) was not altered in co-infected mice. In addition, the relative abundance of AT2 cells in adult mice did not differ between the treatment groups following co-infection during infancy. The strain of virus used in this study is considered to be moderately virulent, hence may not necessarily promote long term damage as seen with more virulent pandemic strains. Alternately, upper airway colonisation requires adhesion to the epithelial lining of the respiratory tract via binding to cell-surface carbohydrates. Increased upper airway carriage in co-infected mice may disrupt the barrier function of polarized upper airway epithelial cells to facilitate greater transmigration of allergens.

SP displays significant genetic diversity with over 90 serotypes and commonly inhabits the upper airways where it can lead to persistent carriage, particularly in infants where colonisation rates in excess of 60% have been reported^22^. Although there are multiple risk factors for impaired SP clearance, co-existence of respiratory viruses markedly increase pneumococcal load during the acute phase of infection and also facilitates transmission^7^. Here, we show in an infant model of co-infection, that IAV facilitates long term nasopharyngeal colonisation into adulthood, in contrast to mice inoculated with SP alone, where the pneumococcus was cleared by day 40. Pneumococcal infection is associated with a robust acute inflammatory response, and clearance of SP is dependent on recruitment of leukocytes to the airways and engagement of humoral-mediated immunity^23^. It has been established that during co-infection with IAV and SP, interferon-dependent anti-viral responses can alter acute cell-mediated immunity to SP leading to a marked increase in pneumococcal load^24^. Here, we show that infant IAV infection has long lasting consequences on pneumococcal colonisation of the upper airways into adulthood.

Acute inflammatory responses to IAV are also known to transiently alter lung function. Interestingly, mice deficient in neutrophils display impaired lung function associated with an increase in airway resistance, tissue damping and tissue elastance as a consequence of impaired viral clearance and more severe histopathology^25^. Hence, an appropriate acute inflammatory response can prevent significant structural changes underlying respiratory functional abnormalities. Infection of neutropenic mice was also characterized by an increase in lung oedema^25^. Consistent with this study, an increase in permeability of the alveolar–capillary barrier was related to AHR and this response was shown to be transient in adult mice as lung function normalised with resolution of inflammation, oedema and tissue repair^13^. In our study, inflammation had resolved with the exception of a small but significant increase in BAL lymphocytes in co-infected mice. The increase in lymphocytes was not associated with an increase in IL-5 or IL-13 expression; hence co-infection did not induce a classic T_H_2 response. SP is known to induce expansion of multiple T lymphocyte subsets including mucosal innate T cells that contribute to control of pneumococcus, and our data suggest that co-infection with IAV promotes lymphocyte accumulation. It has been previously shown that bacterial components of SP can initiate the accumulation of T regulatory cells, which suppress allergic airways disease in response to ovalbumin sensitisation and challenge^26^. In our model, the presence of SP did not confer protection to AHR, possibly as an allergic adjuvant was not used to initiate lung function abnormalities.

Prospective childhood cohorts have now identified viral respiratory infections as a major risk factor for developing persistent wheeze and asthma in individuals with co-existing aeroallergen sensitization^27^. Post natal lung growth represents an important susceptibility period where the combination of viral infections and aeroallergen sensitisation in children can lead to persistent pathological changes and respiratory dysfunction^28^. The role of co-infection in this context is less clear; however there is emerging data to suggest that nasopharyngeal colonisation may increase risk of developing asthma. In a recent study, the presence of bacterial pathogens including SP in the nasopharyngeal compartment during infancy was identified as significant risk determinant for febrile lower respiratory infections and later asthma development^29^.

Hence, it is plausible that prevention of bacterial colonisation in infancy can contribute to the prevention of lower respiratory tract infections and persistent wheeze. This remains a challenging proposition using current strategies as a potential causal link between the use of antibiotics and development of asthma was identified, where disruption of the upper airway microbiome selects for resistant strains that are potentially more pathogenic^29^. The pneumococcal vaccine can reduce risk; however, the niche created by eliminating vaccine-targeted serotypes can be replaced by other serotypes and pathogenic bacteria.

In summary, co-infection caused a significant increase in central airway resistance that was not associated with conventional airway remodelling that underlies AHR to nebulised MCh such as mucus overproduction, smooth muscle thickening or epithelial leakage. An increase in hysteresivity was observed in both IAV and co-infected mice. Hence, co-infection in infancy may be causing distinct structural changes that contribute to ventilation heterogeneity, which persists into adulthood. Lung hysteresivity is a term used to reflect the mechanical coupling between energy dissipative forces and tissue-elastic properties in the parenchyma. Remodelling of the tissue increases hysteresivity in both emphysema and fibrosis in a way that correlates with the volume proportion of collagen, however the physical basis of this phenomenon is not well understood^30^. Our data suggests that there may be microstructural changes contributing to ventilation heterogeneity. Future studies should investigate the impact of co-infection on extracellular matrix components that contribute to the parenchymal architecture and also assess the consequences of combining infant co-infection with aeroallergen sensitisation.

## Additional Information

**How to cite this article**: FitzPatrick, M. *et al*. Neonatal pneumococcal colonisation caused by Influenza A infection alters lung function in adult mice. *Sci. Rep.* **6**, 22751; doi: 10.1038/srep22751 (2016).

## Supplementary Material

Supplementary Information
