# Supplementary material for: Neonatal pneumococcal colonisation caused by Influenza A infection alters lung function in adult mice
Source: Sci Rep. 2016 Mar 4;6:22751. doi: 10.1038/srep22751 (PMC4778168; doi:10.1038/srep22751)
Supplement: Supplementary Information [file srep22751-s1.pdf]

Supplementary information

Neonatal pneumococcal colonisation caused by Influenza A infection alters lung function in adult mice.

Meaghan FitzPatrick[2], Simon G. Royce[5], Shenna Langenbach[2], Jonathan McQualter[2], Patrick C. Reading[3], Odilia Wijburg[4], Gary P. Anderson[2], Alastair Stewart[2], Jane Bourke[2, 5], Steven Bozinovski[1,2,*]

Supplementary methods

Flow cytometric analysis of lung epithelial cells

Single lung cells were isolated by disaggregation of lung tissue by collagenase digestion (Liberase TM; Roche; 1 Wunsch U/3mL Hank's buffered saline solution (HBSS)/lung for 45 min at 37°C with agitation) followed by hypotonic lysis of red blood cells (10mM $KHCO_3$, 150mM NH_4Cl, 0.1mM EDTA Na_2 for 90 sec at RT). Single cells were then resuspended at 5 x 10^7 cells/mL in HBSS plus 2% (v/v) fetal bovine serum (FBS; Invitrogen) containing a cocktail of antibodies (anti-CD45, anti-CD31, anti-EpCAM, anti-MHCII) and relevant isotype controls (Biolegend). Labeled cells were washed in HBSS-2% FBS, resuspended at 1 x 10^7 cells/mL and held on ice for flow cytometric analysis. Viability was determined by propidium iodide (PI; 1 µg/mL) staining, and doublets excluded by forward/side scatter (height) vs. forward/side scatter (width) gating. Type II alveolar (AT2) cells were identified by the phenotype $CD45^-$ $CD31^-$ $EpCAM^+$ $MHCII^+$.

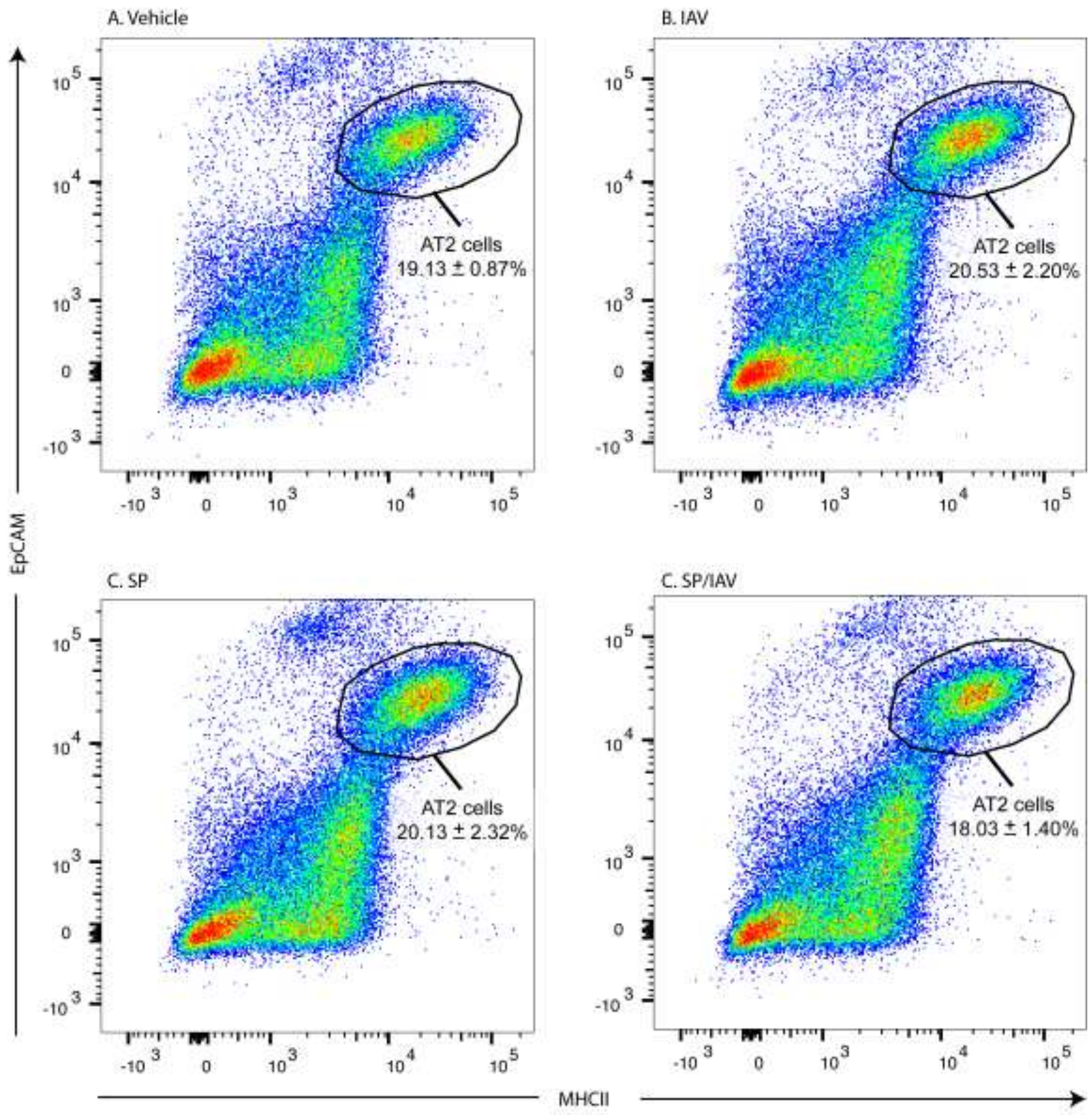

Figure S1. The relative abundance of At2 cells does not alter between groups. Representative images of FACS plots of viable, non-hematopoietic ($CD45^-$), non-endothelial ($CD31^-$) cells from PBS vehicle (A), IAV (B), SP (C) and SP/IAV treated (D) mice shows no difference in the relative abundance of $EpCAM^+$ $MHCII^+$ AT2 cells between groups. Quantification is shown as mean ± sem (n=3).

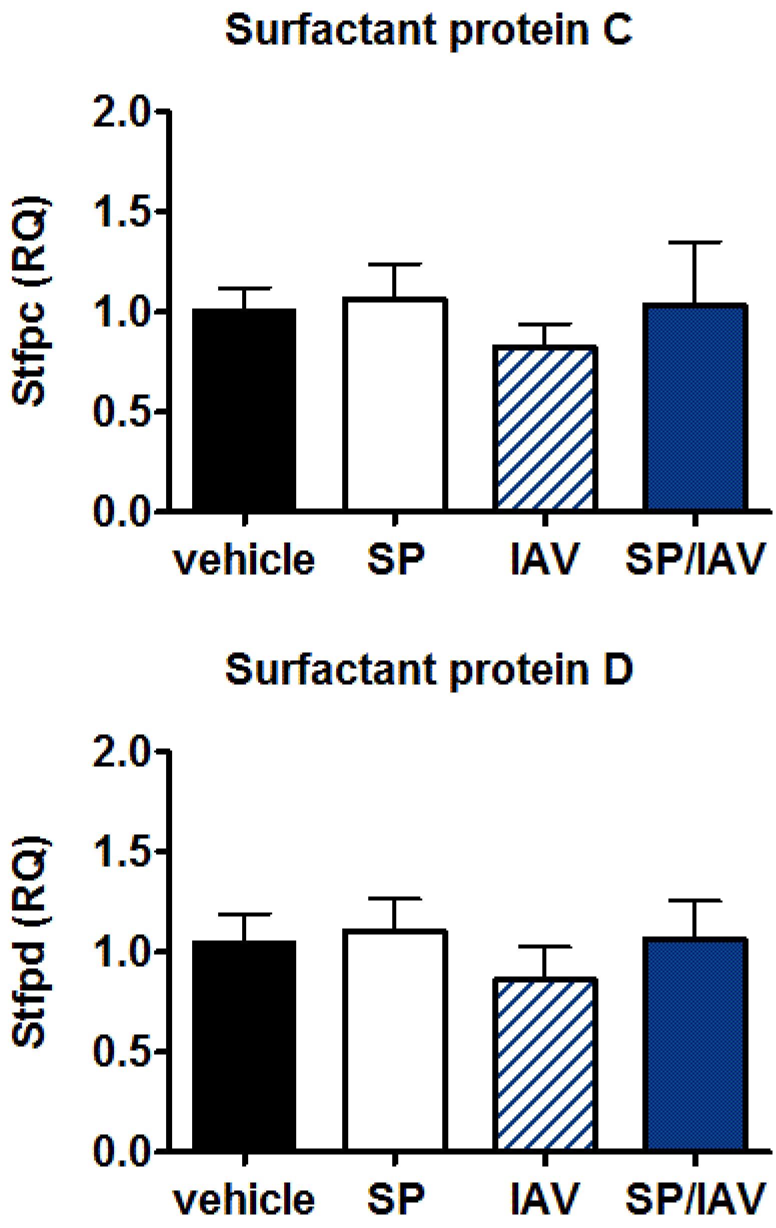

Figure S2. Neonatal co-infection does not alter surfactant protein gene expression in adult lung Taqman PCR analysis for gene expression of surfactant protein C and D in lung tissue is expressed as fold-change compared to vehicle-treated control and normalised to GAPDH housekeeping gene (n = 6).
